# Monocyte/Lymphocyte Ratio and Cardiovascular Disease Mortality in Peritoneal Dialysis Patients

**DOI:** 10.1155/2020/9852507

**Published:** 2020-02-14

**Authors:** Yueqiang Wen, Xiaojiang Zhan, Niansong Wang, FenFen Peng, Xiaoran Feng, Xianfeng Wu

**Affiliations:** ^1^Department of Nephrology, The Second Affiliated Hospital of Guangzhou Medical University, Guangzhou, China; ^2^Department of Nephrology, The First Affiliated Hospital of Nanchang University, Nanchang, China; ^3^Department of Nephrology, Affiliated Sixth People's Hospital, Shanghai Jiao Tong University, Shanghai, China; ^4^Department of Nephrology, Zhujiang Hospital of Southern Medical University, Guangzhou, China; ^5^Department of Nephrology, Jiujiang No. 1 People's Hospital, Jiujiang, China

## Abstract

**Objectives:**

The monocyte-to-lymphocyte ratio (MLR), as a new marker of the systemic inflammatory response, is associated with cardiovascular disease (CVD) mortality in the general population and hemodialysis patients. However, the association between the MLR and CVD mortality in peritoneal dialysis (PD) has received little attention.

**Methods:**

In this multicenter retrospective cohort study, 1753 incident PD patients from November 1, 2005, to June 30, 2017, with a baseline MLR were enrolled. The primary endpoint was CVD mortality. The association of MLR with CVD mortality was assessed using a multivariable-adjusted Cox model and the Fine and Gray competing risk model.

**Results:**

Of 1753 patients, the mean age was 51.1 ± 14.9 years, 56.9% of patients were male, and the Charlson comorbidity index was 4.29 ± 1.75. During the follow-up period of 31.2 ± 18.4 months, 368 patients died, of which 200 (54.3%) deaths were caused by CVD events. CVD mortality rates for the lowest, middle, and highest MLR tertiles were 70.6, 78.4, and 88.9 per 1000 patient-years, respectively (*P* < 0.001). Kaplan-Meier analysis revealed that survival rates were significantly different among the three MLR groups (log rank = 22.41, *P* < 0.001). Kaplan-Meier analysis revealed that survival rates were significantly different among the three MLR groups (log rank = 22.41, *P* < 0.001). Kaplan-Meier analysis revealed that survival rates were significantly different among the three MLR groups (log rank = 22.41, *P* < 0.001). Kaplan-Meier analysis revealed that survival rates were significantly different among the three MLR groups (log rank = 22.41, *P* < 0.001). After adjusting for confounding factors, the highest MLR tertile was significantly associated with a hazard ratio (HR) for CVD mortality of 1.45 (95% confidence interval, 1.13-2.51, *P* = 0.016). The Fine and Gray method analysis showed that using all-cause mortality as competing risk, the highest MLR tertile remained an independent predictor of CVD mortality (HR = 1.39, 95% CI 1.10-2.47, *P* = 0.021).

**Conclusions:**

Higher MLR levels at the commencement of PD may be independently associated with increased CVD mortality in PD patients.

## 1. Introduction

Cardiovascular disease (CVD) is a severe complication of peritoneal dialysis (PD) and is to blame for nearly 60% of all-cause deaths in this population [1]. The high CVD mortality is not entirely explained by traditional cardiovascular risk factors [2]. Beyond traditional risk factors such as diabetes and hypertension, uremia-specific factors that arise from accumulating toxins also contribute to the pathogenesis of CVD [2]. Inflammation, as one of the well-recognized nontraditional risk factors, contributes to excessive CVD mortality in PD patients [3]. Among PD patients, chronic low-grade inflammation is a critical factor in malnutrition inflammation-atherosclerosis/calcification syndrome, which further accelerates the progression of atherosclerosis and is associated with an increased risk of CVD [4].

Compared with traditional inflammation markers such as tumor necrosis factor-alpha (TNF-a) and interleukin-6 (IL-6), the neutrophil-to-lymphocyte ratio (NLR) and monocyte-to-lymphocyte ratio (MLR) are new, inexpensive, and reproducible markers of the systemic inflammatory response that are suitable for routine use and can be easily calculated from a white blood cell assay and determined under simple laboratory conditions [5–8]. Previous studies reported that the NLR is an independent factor of clinical outcomes in coronary artery disease and an attractive biomarker for predicting the severity of the lesion, which has the potential to be a universal biomarker in clinical applications [9–12]. The MLR has been proven to be a prognostic factor in patients with malignancies and tuberculosis [13, 14]. More recently, a study found that the MLR was an independent risk factor for the presence of CVD and a predictor of lesion severity in patients with previous coronary artery disease [5]. Another study from China found that a higher MLR was a strong and independent predictor of all-cause and CVD mortality among Chinese hemodialysis patients [15]. However, the association between MLR and CVD mortality remains unknown in PD patients. Therefore, in the present study, we aimed to assess the association between MLR and CVD mortality in PD patients.

## 2. Materials and Methods

### 2.1. Study Design and Population

We study all incident PD patients who were followed up at PD centers in China from November 1, 2005, to February 28, 2017. Inclusion criteria were age ≥ 18 years at the start of PD and survival for ≥3 months from the first PD therapy. Patients were excluded from the study if they had been diagnosed with hematological diseases, active malignancies, or rheumatic diseases or were using any corticosteroids or immunosuppressants. Patients with acute infection were treated and then enrolled in the study. The study was consistent with the ethical principles of the Declaration of Helsinki and approved by the Research Ethical Committee of each study organization. Written informed consent was signed by PD patients when they started to receive PD treatment.

Baseline demographic data included age, sex, hypertension, a history of CVD, diabetes, Charlson comorbidity index (CCI), current smoking and drinking, and medication use. Clinical and biochemical data at the initiation of PD included body mass index (BMI), systolic blood pressure (BP), diastolic BP, 24 h urine output, monocytes, lymphocytes, serum albumin, total cholesterol, triglycerides, low-density lipoprotein (LDL), high-density lipoprotein (HDL), serum uric acid, high-sensitivity C-reactive protein (hs-CRP), N-terminal probrain natriuretic peptide (NT-pro-BNP), and estimated glomerular filtration rate (eGFR). All baseline data were obtained during the first month of PD.

The primary endpoint was CVD mortality, which was defined as death caused by coronary events, arrhythmias, sudden cardiac death, congestive heart failure, or cerebrovascular events [16]. The PD team consisted of two nephrologists at our centers who reviewed the details of individual medical records and identified the causes of death. If death had two or more potential causes, we generally ascribed the death to the primary cause for hospitalization or the initial presenting condition. If a patient died within three months of transfer to hemodialysis therapy, he or she was not censored because the early mortality was considered to reflect health status during the period of failing PD treatment. All patients were followed up until cessation of PD, death, or May 31, 2017. The censored data included switching to HD, renal transplantation, moving to another center, loss to follow-up, or being still at our PD centers. Because 259 (14.8%) PD patients have survived more than five years, in the present study, we only evaluated the association between the monocyte/lymphocyte ratio and 5-year risk of CVD mortality in PD patients. Thus, even though PD patients have survived more than five years, we only collected data for five years. All patients received continuous ambulatory PD treatment. Conventional PD solutions (Dianeal 1.5%, 2.5%, or 4.25% dextrose; Baxter Healthcare, Guangzhou, China), Y sets, and twin bag systems were used in all PD patients. The comorbidity score was determined according to the CCI, which is one of the most commonly used comorbidity models [17]. Baseline residual renal function was assessed by eGFR using the Chronic Kidney Disease Epidemiology Collaboration creatinine equation [18].

### 2.2. Statistical Analyses

Eligible patients were divided into three groups by the tertile value of the MLR. The lowest MLR tertile had an MLR value < 0.29, the middle MLR tertile had an MLR value of 0.29 to 0.45, and the highest MLR tertile had an MLR value > 0.45. Data were expressed as percentages, mean ± standard deviation, or median (25^th^-75^th^ percentile). Continuous variables were compared by employing one-way ANOVA or the Kruskal-Wallis test, and categorical variables were tested using the *χ*^2^ test among the three groups. The correlations between baseline variables and MLR were assessed by correlation analysis. Multivariate linear regressions were performed to identify the independent determinants of MLR. Survival was estimated using the Kaplan-Meier curve, and differences were examined using the log-rank test. The associations between the MLR tertiles and outcomes were evaluated by Cox proportional hazards regression. Unadjusted associations were first examined, followed by adjustments for age, sex, CCI, current smoking and drinking, and medication use, including angiotensin-converting enzyme inhibitors/angiotensin receptor blockers (ACEIs/ARBs), *β*-blockers, and statins. Next, albumin, cholesterol, triglycerides, LDL, HDL, serum uric acid, hs-CRP, NT-pro-BNP, and eGFR were added to examine whether the association of the MLR tertiles with CVD mortality was independent of confounding factors. In the present study, comorbidities such as diabetes were included in the CCI, which was included in multivariable Cox models. Therefore, to reduce the effect of the interaction between comorbidities and the CCI on outcomes, we only included the CCI in multivariable Cox models.

### 2.3. Sensitivity Analysis

All-cause mortality and transplantation or hemodialysis were considered competing risks. The covariates included in the three Cox regression models were evaluated by the Fine and Gray competing risk model [19]. The results of the Cox analysis and the Fine and Gray model were presented as the hazard ratio (HR) and the 95% confidence interval (CI).

### 2.4. Subgroup Analyses

We firstly analyzed the difference of CVD mortality rates among MLR tertiles in subgroups, including male, female, diabetes, nondiabetes, those with a history of CVD, those without a history of CVD, hypertension, and nonhypertension subgroups. Those subgroups, in which CVD mortality rates had significant differences among MLR tertiles, were further analyzed using survival analysis, a life table, Cox regression, and the Fine and Gray models.

A value of *P* < 0.05 was considered statistically significant. Statistical analyses were performed using GraphPad Software 8.0 (GraphPad Prism Software Inc., San Diego, California) and the R package 3.6.0 (https://www.r-project.org/).

## 3. Results

### 3.1. Baseline Characteristics

**Figure 1:**
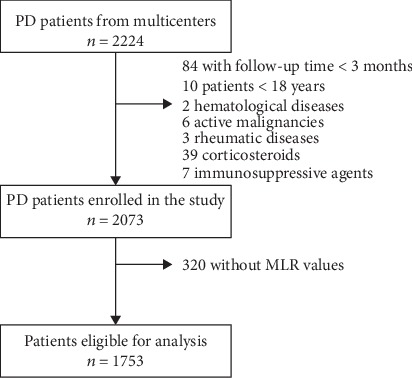
The flow chart shows how patients were selected for the present study. PD: peritoneal dialysis; MLR: monocyte-to-lymphocyte ratio.

A total of 2224 incident PD patients were enrolled at these PD centers, of whom 10 patients who were younger than 18 years, 84 patients who were on PD<3 months, 2 patients who have hematological diseases, 6 patients who have active malignancies, 3 patients who have rheumatic diseases, 39 patients who used corticosteroids, 7 patients who used immunosuppressants, and 320 patients who lacked serum MLR values at baseline were excluded. The remaining 1753 patients with MLR values measured at baseline were eligible for the present analysis (Figure 1). Of 1753 patients, the mean age was 51.1 ± 14.9 years, 56.9% of patients were male, 18.8% patients had a history of CVD, 23.8% patients had diabetes mellitus, and 71.0% patients had hypertension. The CCI was 4.29 ± 1.75.

The baseline characteristics of the study population are shown in Table 1. The baseline characteristics were compared among the three MLR tertiles, except for age, sex, and CCI. Patients in the highest MLR tertile were older (*P* < 0.001), were more likely to be male sex (*P* < 0.001), and had higher CCI (*P* < 0.001) than those in the lowest MLR tertile.

### 3.2. Variables Relevant to the MLR Values

Univariate analysis identified age (*P* < 0.001), diabetes (*P* = 0.015), and CCI (*P* < 0.001) as variables that were positively related to the MLR, while serum albumin (*P* = 0.017) was negatively related to the MLR. Multivariate analysis considering associated variables retained the correlation of the MLR with age (*P* < 0.001) and serum albumin (*P* = 0.021).

### 3.3. MLR Tertiles and CVD Mortality

**Figure 2:**
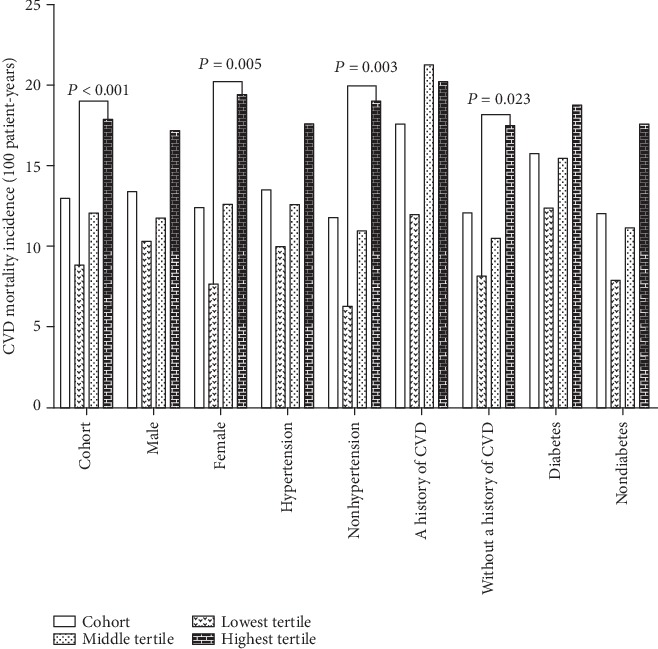
Cumulative CVD mortality of patients in different MLR tertiles. CVD: cardiovascular disease.

**Figure 3:**
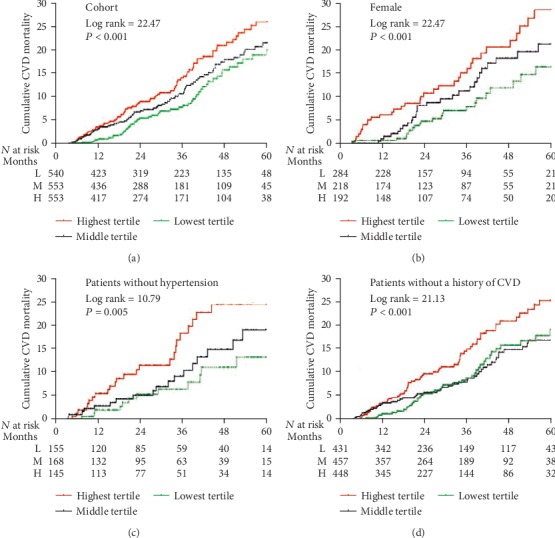
Cumulative CVD mortality curves for patients in different MLR tertiles. CVD: cardiovascular disease.

The median follow-up period was 31.2 ± 18.4 months. By the end of this study, 368 (21.0%) patients had died, 103 (5.9%) patients had undergone kidney transplantation, 225 (12.8%) patients had transferred to HD, 22 (1.3%) patients had transferred to other PD centers, and 34 (1.9%) patients had been lost to follow-up; the remaining 1001 (57.1%) patients were still followed at these PD centers. Of 368 deaths, 200 (54.3%) deaths were caused by CVD episodes. The CVD mortality rates for the lowest, middle, and highest MLR tertiles were 70.6, 78.4, and 88.9 per 1000 patient-years, respectively (Figure 2). There were significant differences in the CVD mortality rates in these three groups (*P* < 0.001). The Kaplan-Meier estimates of the CVD mortality for patients with different MLR tertiles are shown in Figure 3. The Kaplan-Meier estimates revealed that the survival rates were significantly different among the three MLR tertiles in the cohort study (log rank = 22.47, *P* < 0.001, Figure 3(a)). At the end of 1, 3, and 5 years in this cohort, CVD mortality was, respectively, 5.7%, 15.6%, and 19.8% in the lowest MLR tertile; 6.8%, 16.9%, and 20.6% in the middle MLR tertile; and 9.9%, 21.6%, and 26.7% in the highest MLR tertile. The association between the baseline MLR tertiles and CVD mortality is shown in Table 2. Crude Cox model analysis showed that an increased MLR was a significant predictor of CVD mortality (HR = 1.64, 95% CI 1.16-2.32, *P* = 0.005, model 1). Multivariate Cox model analysis found that an increased MLR was an independent predictor of CVD mortality, even after adjusting for age, sex, CCI, current smoking and drinking, medication use, serum albumin, total cholesterol, triglycerides, LDL, HDL, serum uric acid, hs-CRP, NT-pro-BNP, and eGFR (HR = 1.45, 95% CI 1.13-2.51, *P* = 0.016, model 3).

### 3.4. Sensitivity Analyses

With all-cause mortality as a competing risk event, the highest MLR tertile had an HR for CVD mortality of 1.39 (95% CI 1.10-2.47, *P* = 0.021) compared to the lowest MLR tertile, after adjusting for confounding factors (Table 2). Besides, with renal transplantation or hemodialysis as a competing risk event, the highest MLR tertile remained an independent predictor for all-cause mortality (HR = 1.37, 95% CI 1.09-2.54, *P* = 0.026) compared to the lowest MLR tertile, after adjusting for confounding factors.

### 3.5. Subgroup Analyses

**Figure 4:**
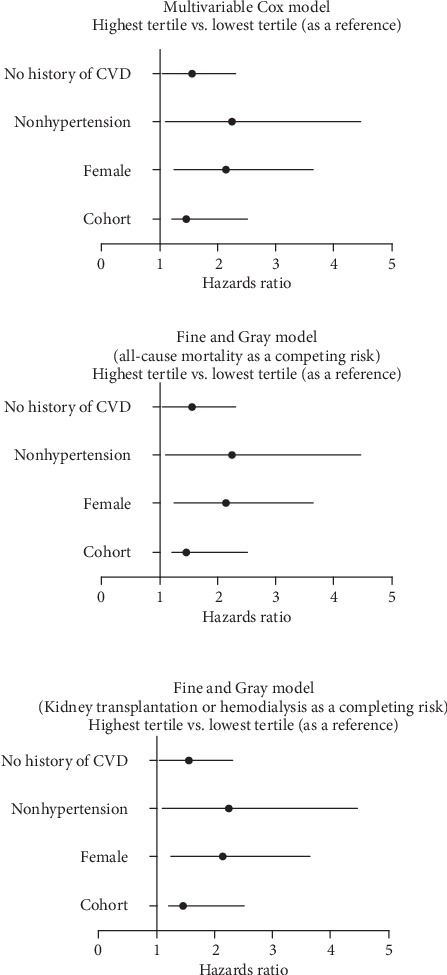
Adjusted hazard ratios for CVD mortality. Hazard ratios for the highest MLR tertile relative to the lowest MLR tertile (adjusted in multivariable models). CVD: cardiovascular disease.

It is worth noting that the CVD mortality rate in the highest MLR tertile in patients with a history of CVD (118.0/1000 patient-years) seemed to be higher as compared to the other subgroups (ranged from 67.9 to 95.6/1000 patient-years, Figure 2). There were significant differences in CVD mortality rates among the three MLR tertiles in three subgroups, including female (*P* = 0.005), nonhypertension (*P* = 0.033), and those without a history of CVD subgroups (*P* = 0.023), whereas these trends were not observed among those male, hypertension, a history of CVD or diabetes, and nondiabetes subgroups. Thus, the female, nonhypertension, and those without a history of CVD subgroups were further analyzed. The Kaplan-Meier estimates of the cumulative CVD mortality in different MLR tertiles were evaluated among three subgroups (Figures 3(b)–3(d)). CVD mortality at 1, 3, and 5 years in three subgroups are shown in Table 3. MLR tertiles were associated with CVD mortality among the female, hypertension, and those without a history of CVD subgroups. Using the Cox analysis and the Fine and Gray competing risk model, adjusted HRs for CVD mortality among three subgroups are shown in Figure 4.

## 4. Discussion

Our results showed that the baseline MLR values were independently associated with increased risk of CVD mortality in PD patients. Even using all-cause mortality and kidney transplantation or hemodialysis as competing risks, the higher MLR values remained an independent predictor for CVD mortality. Subgroup analyses found that similar trends were observed in the female, nonhypertension, and those without a history of CVD subgroups. In addition, patients with higher baseline MLR values may be elderly and more likely to be male and have higher CCI.

PD patients are at 5- to 10-fold higher risk for developing CVD than the age-matched general population [4]. Among PD patients, CVD is the main complication and major cause of death, accounting for nearly 60% of all-cause deaths [1]. However, this excessive CVD mortality is unlikely to be fully accounted for by traditional Framingham risk factors (age, male sex, diabetes, hypertension, hyperlipidemia, and smoking) because advances in relevant treatment fail to improve CVD risk significantly in ESRD patients [3]. Nontraditional Framingham risk factors, including inflammation, anemia, and abnormal bone and mineral metabolism, should be focused on PD patients [20]. It is worth noting that systemic inflammation is now recognized as one of the critical components in atherosclerosis in the general population and may accelerate atherosclerosis in ESRD patients [15]. The estimated prevalence of systemic inflammation ranged between 12% and 65% in PD patients, and systemic inflammation was associated with significant CVD and all-cause death [21, 22].

The NLR and MLR, novel inflammatory markers, have a better kinetic pattern than the traditional inflammatory marker, hs-CRP distribution [23]. The NLR and MLR reflect two immune pathways, which are probably less affected by confounding conditions and may be more predictive in evaluating inflammation than neutrophils, monocytes, platelets, or lymphocytes separately [24, 25]. Recent studies demonstrate that the NLR has been shown to predict CVD and all-cause mortality in the general population and ESRD patients [26]. A study focusing on 260 HD patients reported that patients with a higher NLR had a lower survival at the end of the study, and a high NLR was an independent predictor of all-cause and CVD mortality when adjusted for other risk factors [26, 27]. Another study with 86 PD patients found that patients with a higher NLR had a lower survival during follow-up, and Kaplan-Meier curves showed that the cumulative incidences of both CVD mortality and all-cause mortality were significantly higher in patients with a higher NLR [27]. In addition, previous studies have found that the MLR has been shown to predict CVD and all-cause mortality in the general population and in HD patients [5, 15]. A recent study of 543 patients undergoing coronary angiography reported that an increased MLR was an independent risk factor for the presence of coronary artery disease (odds ratio (OR) = 3.94, 95% CI: 1.20–12.95) and a predictor of lesion severity (OR = 2.05, 95% CI: 1.15–3.66) [5]. Another study, a single-center study with 355 HD patients, found that an increased MLR showed lower survival rates compared with a lower MLR using Kaplan-Meier analysis (*P* < 0.001), and the MLR was independently associated with CVD mortality in multivariate analyses (HR = 6.985, 95% CI 1.943–25.115, *P* = 0.003) [15]. However, the association between the MLR and CVD mortality in PD patients remains unknown. In the present study, we found that higher MLR values were positively related to age and CCI but were negatively related to serum albumin. Notably, a higher MLR was not related to hs-CRP, which was inconsistent with results from a previous study. This study reported that the MLR was positively related to hs-CRP [15]. In addition, multivariable Cox regression identified that the highest MLR was significantly associated with an HR of 1.45 for CVD mortality, even after adjusting for age, sex, CCI, current smoking and drinking, medication use, serum albumin, total cholesterol, triglycerides, LDL, HDL, serum uric acid, hs-CRP, NT-pro-BNP, and eGFR in our study. To reduce the effect of confounding on the results in the present study, we further analyzed the association between baseline MLR and CVD mortality using a competing risk model. With all-cause mortality and transplantation or hemodialysis as competing risks, the baseline higher MLR values remained an independent predictor for CVD mortality.

Further subgroup analyses using Kaplan-Meier analysis revealed that a higher MLR conferred a higher risk of cumulative CVD mortality in female patients and those without hypertension or a history of CVD. The reasons for the above results were that female patients were less likely to have diabetes, a history of CVD, and hypertension and had lower CCI; those without a history of CVD were less likely to be older and have diabetes and hypertension and had lower CCI; and those without hypertension were less likely to be older and have diabetes and a history of CVD and had lower CCI (data not shown). These reasons may reduce the interaction between the baseline MLR and other confounding factors in CVD mortality, which may highlight the effect of the MLR on CVD mortality in PD patients. Besides, it was worth noting that the CVD mortality in those with a history of CVD (118.0/1000 patient-years) with higher MLR values seemed to be the higher CVD mortality compared with the other subgroups (CVD mortality ranged from 67.9 to 95.6/1000 patient-years). This finding suggested that compared with other risk factors for CVD mortality, a history of CVD may play a more prominent role in CVD mortality in PD patients.

There are several limitations to the present study. First, a retrospective study allows us to establish associations but not causal relationships, and we could adjust all factors associated with higher CVD mortality due to the restriction of sample size. The effect of residual confounding, therefore, cannot be eliminated. However, this study was a multicenter study with the ability to adjust for significant risk factors for CVD mortality, as well as use all-cause mortality and kidney transplantation or hemodialysis as competing risks of CVD mortality. Besides, baseline characteristics among the three MLR tertiles were compared except for age, sex, and CCI. Thus, the independent association between the MLR and CVD mortality may be convincing in the present study. Second, our study lacks information on several traditional inflammation markers, such as TNF-a and IL-6. Compared with novel inflammatory markers such as the NLR or MLR, TNF-a and IL-6 measurements are more expensive and inconvenient, and they are not measured routinely because the assay is not readily available and is expensive. In their routine follow-up surveillance, PD patients need less expensive and more convenient measurements of inflammatory markers. Therefore, the MLR may be a suitable surrogate for TNF-a and IL-6 for PD patients concerning results from the present study and previous studies. Third, we assessed only baseline variables rather than changes over time in these variables of CVD mortality. Finally, our study participants were all Chinese; therefore, our results may not apply to other ethnic groups.

## 5. Conclusions

We found an independent association between high MLR values and increased CVD mortality in PD patients, especially in females and those without hypertension or a history of CVD. These findings suggested, along with previous studies, that PD patients with a higher MLR may have more CVD involvement and a preprocedural MLR, a widely available, inexpensive, and inflammatory biomarker, could be helpful for risk stratification of CVD mortality.

## Figures and Tables

**Table 1:** Baseline characteristics of the study population.

Variables	Lowest MLR tertile	Middle MLR tertile	Highest MLR tertile	*P* value
	MLR < 0.29 (*n* = 583)	MLR 0.29-0.45 (*n* = 585)	MLR > 0.45 (*n* = 585)	
Age (years)	49.6 ± 14.9	49.8 ± 14.7	53.8 ± 14.8	<0.001
Male sex (%)	272 (46.7)	346 (59.1)	379 (64.8)	<0.001
Hypertension (%)	408 (0.70)	404 (69.1)	432 (73.8)	0.160
A history of CVD (%)	108 (18.5)	89 (15.2)	104 (17.8)	0.290
Diabetes (%)	129 (22.1)	129 (22.1)	160 (27.4)	0.051
CCI	4.16 ± 1.75	4.09 ± 1.69	4.62 ± 1.78	<0.001
Current smoking (%)	26 (4.5)	32 (5.5)	29 (5.0)	0.729
Current drinking (%)	5 (0.9)	12 (2.1)	8 (1.4)	0.225
ACEI/ARB use (%)	195 (33.0)	195 (33.0)	213 (36.4)	0.455
*β*-Blocker use (%)	134 (23.0)	112 (19.1)	140 (23.9)	0.112
Statin use (%)	69 (11.8)	72 (12.3)	94 (16.1)	0.067
BMI (kg/m^2^)	22.3 ± 3.6	22.3 ± 3.2	22.2 ± 3.7	0.906
Systolic BP (mmHg)	150 ± 26	148 ± 27	149 ± 25	0.477
Diastolic BP (mmHg)	88 ± 15	87 ± 16	87 ± 15	0.778
24 h urine output (ml)	600 (200-1050)	700 (300-1100)	650 (200-1000)	0.062
Monocytes (∗10^9^/l)	0.47 ± 0.24	0.45 ± 0.23	0.50 ± 0.25	0.003
Lymphocytes (∗10^9^/l)	1.30 ± 0.52	1.28 ± 0.52	1.20 ± 0.48	0.005
Albumin (g/dl)	3.35 ± 0.78	3.39 ± 0.72	3.30 ± 0.67	0.107
Cholesterol (mg/dl)	151 ± 67	152 ± 62	156 ± 66	0.421
Triglycerides (mg/dl)	125 ± 99	130 ± 99	127 ± 89	0.686
LDL (mg/dl)	2.55 ± 1.02	2.52 ± 0.95	2.60 ± 1.03	0.506
HDL (mg/dl)	1.14 ± 0.39	1.14 ± 0.38	1.13 ± 0.37	0.411
Serum uric acid (mg/dl)	7.06 ± 2.42	7.34 ± 2.35	7.14 ± 2.42	0.118
hs-CRP (mg/l)	21.8 (5.40-82.7)	9.9 (2.4-37.2)	6.6 (2.3-22.6)	0.746
NT-pro-BNP (pg/ml)	3207 (1141-8789)	2810 (504-14675)	2135 (1034-5757)	0.464
eGFR (ml/min/1.73 m^2^)	5.71 (4.31–8.40)	5.69 (4.29–8.64)	5.68 (4.23–7.44)	0.367

MLR: monocyte-to-lymphocyte ratio; CVD: cardiovascular disease; CCI: Charlson comorbidity index; ACEI/ARB: angiotensin-converting enzyme inhibitor/angiotensin receptor blocker; BP: blood pressure; LDL: low-density lipoprotein; HDL: high-density lipoprotein; hs-CRP: high-sensitivity C-reactive protein; NT-pro-BNP: N-terminal probrain natriuretic peptide; eGFR: estimated glomerular filtration rate.

**Table 2:** Association between the baseline MLR tertiles and CVD mortality.

MLR	Model 1		Model 2		Model 3	
	HR (95% CI)	*P*	HR (95% CI)	*P*	HR (95% CI)	*P*
Cox model						
Lowest tertile	1.0		1.0		1.0	
Middle tertile	1.24 (1.13-2.34)	0.013	1.19 (1.09-2.46)	0.030	1.13 (1.07-2.55)	0.037
Highest tertile	1.64 (1.16-2.32)	0.005	1.56 (1.15-2.44)	0.009	1.45 (1.13-2.51)	0.016
Competing risk^∗^						
Lowest tertile	1.0		1.0		1.0	
Middle tertile	1.22 (1.11-2.33)	0.014	1.16 (1.08-2.40)	0.032	1.10 (1.06-2.50)	0.039
Highest tertile	1.61 (1.15-2.30)	0.007	1.52 (1.14-2.43)	0.011	1.39 (1.10-2.47)	0.021
Competing risk^#^						
Lowest tertile	1.0		1.0		1.0	
Middle tertile	1.18 (1.09-2.40)	0.017	1.14 (1.07-2.46)	0.037	1.09 (1.05-2.56)	0.040
Highest tertile	1.57 (1.13-2.36)	0.010	1.48 (1.11-2.49)	0.018	1.37 (1.09-2.54)	0.026

^∗^All-cause mortality as a competing risk. ^#^Kidney transplantation or hemodialysis as a competing event risk. Model 1: unadjusted. Model 2: adjusted for age, sex, CCI, current smoking, current drinking, ACEI/ARB use, *β*-blocker use, and statin use. Model 3: model 2 adjusted for serum albumin, total cholesterol, triglycerides, LDL, HDL, serum uric acid, hs-CRP, NT-pro-BNP, and eGFR. CVD: cardiovascular disease; MLR: monocyte-to-lymphocyte ratio; CCI: Charlson comorbidity index; ACEI/ARB: angiotensin-converting enzyme inhibitor/angiotensin receptor blocker; HDL: high-density lipoprotein; hs-CRP: high-sensitivity C-reactive protein; NT-pro-BNP: N-terminal probrain natriuretic peptide; eGFR: estimated glomerular filtration rate.

**Table 3:** CVD mortality at 1, 3, and 5 years in three subgroups.

	1 year	3 years	5 years
Female sex			
Lowest tertile	4.6%	12.1%	16.9%
Middle tertile	7.8%	18.1%	21.1%
Highest tertile	10.7%	21.7%	28.7%
Patients without hypertension			
Lowest tertile	5.3%	11.2%	13.4%
Middle tertile	5.2%	14.9%	19.3%
Highest tertile	11.4%	17.3%	23.7%
Patients without a history of CVD			
Lowest tertile	5.6%	15.8%	18.7%
Middle tertile	5.9%	14.9%	17.1%
Highest tertile	9.8%	20.7%	25.1%

CVD: cardiovascular disease.

## Data Availability

The data used to support the findings of this study are available from the corresponding author upon request.
